# Lung function variability and the burden of COPD and lung restriction in South Africa and Burkina Faso: results from the AWI-Gen study

**DOI:** 10.64898/2026.09.22.26363668

**Published:** 2026-09-23

**Authors:** Emmanuel Adonyo, Jing Chen, Catherine John, Richard Packer, Michele Ramsay, Chiara Batini, Richard van Zyl-Smit, Mervyn Mer, Palwende Romuald Boua, Godfred Agongo, F Xavier Gómez-Olivé, Reneilwe Given Mashaba, Martin D. Tobin, Anna L. Guyatt, Lisa K. Micklesfield

## Abstract

**Background:**

Population-based spirometry data from sub-Saharan Africa remain limited. We characterised lung function, chronic obstructive pulmonary disease (COPD), lung restriction and associated factors from two Sub-Saharan African countries.

**Methods:**

We performed spirometry using ndd Easy On-PC spirometers among adults aged ≥40 years from population-based cohorts in four communities in Burkina Faso and South Africa from January 2019–April 2022. We included 1,619 participants (936 women, 683 men) with repeatable forced expiratory volume in one second (FEV _1_ ) and forced vital capacity (FVC). COPD was defined as FEV _1_ /FVC<0.70 and lung restriction as FEV _1_ /FVC≥0.70, with FVC<80% predicted. We used multivariable models to test factors associated with lung function measurements (FEV _1_ , FVC and FEV _1_ /FVC), COPD and lung restriction.

**Results:**

Median FEV _1_ and FVC ranged from 1.940–2.273L and 2.521–2.883L, respectively. COPD prevalence was 16.1% (95% CI 14.4–18.0; site range 10.8%–24.5%) and lung restriction prevalence was 21.3% (95% CI 19.4–23.4; site range 11.3%–36.2%). Prior tuberculosis was associated with higher odds of COPD (OR 2.86, 95% CI 1.75–4.59) and lower FEV _1_ , FVC and FEV _1_ /FVC. HIV infection was associated with higher COPD odds (OR 1.67, 95% CI 1.01–2.72) and lower FEV _1_ /FVC. Hypertension was associated with higher lung restriction odds (OR 1.46, 95% CI 1.07–2.00) and lower FEV _1_ and FVC. Diabetes was associated with lower COPD odds (OR 0.50, 95% CI 0.24–0.94), lower FVC and higher FEV _1_ /FVC. Ever-smoking and older age were associated with higher COPD odds (OR 2.21, 95% CI 1.48–3.32; OR 1.05, 95% CI 1.03–1.07, respectively); older age was associated with higher lung restriction odds (OR 1.02, 95% CI 1.00–1.04).

**Conclusion:**

This multisite study highlights substantial heterogeneity in COPD and lung restriction prevalence across African populations and identifies respiratory multimorbidity with infectious and cardiometabolic conditions. These findings provide population-based evidence to inform respiratory surveillance in sub-Saharan Africa.

## Full Text Availability

The license terms selected by the author(s) for this preprint version do not permit archiving in PMC. The full text is available from the preprint server.

